# Collagen scaffold-seeded iTenocytes accelerate the healing and functional recovery of Achilles tendon defects in a rat model

**DOI:** 10.3389/fbioe.2024.1407729

**Published:** 2024-12-06

**Authors:** Thomas Später, Patricia Del Rio, Oksana Shelest, Jacob T. Wechsler, Giselle Kaneda, Melissa Chavez, Julia Sheyn, Victoria Yu, Wolfgang Metzger, Dave Huang, Melodie Metzger, Wafa Tawackoli, Dmitriy Sheyn

**Affiliations:** ^1^ Orthopaedic Stem Cell Research Laboratory, Cedars-Sinai Medical Center, Los Angeles, CA, United States; ^2^ Board of Governors Regenerative Medicine Institute, Cedars-Sinai Medical Center, Los Angeles, CA, United States; ^3^ Department of Trauma, Hand and Reconstructive Surgery, Saarland University, Homburg, Germany; ^4^ Orthopedics Biomechanics Laboratory, Cedars-Sinai Medical Center, Los Angeles, CA, United States; ^5^ Department of Orthopedics, Cedars-Sinai Medical Center, Los Angeles, CA, United States; ^6^ Department of Surgery, Cedars-Sinai Medical Center, Los Angeles, CA, United States; ^7^ Department of Biomedical Sciences, Cedars-Sinai Medical Center, Los Angeles, CA, United States

**Keywords:** tissue regeneration, stem cells, collagen scaffold, Achilles tendon rupture repair, tissue engineering

## Abstract

**Introduction:**

Tendon injuries represent an ongoing challenge in clinical practice due to poor regenerative capacity, structure, and biomechanical function recovery of ruptured tendons. This study is focused on the assessment of a novel strategy to repair ruptured Achilles tendons in a Nude rat model using stem cell-seeded biomaterial.

**Methods:**

Specifically, we have used induced pluripotent stem cell (iPSC)-derived mesenchymal stem cells (iMSCs) overexpressing the early tendon marker Scleraxis (SCX, iMSC^SCX+^, iTenocytes) in combination with an elastic collagen scaffold. Achilles tendon defects in Nude rat models were created by isolating the tendon and excising 3 mm of the midsection. The Achilles tendon defects were then repaired with iTenocyte-seeded scaffolds, unseeded scaffolds, or suture only and compared to native Nude rat tendon tissue using gait analyses, biomechanical testing, histology, and immunohistochemistry.

**Results:**

The results show faster functional recovery of gait in iTenocyte-seeded scaffold group comparing to scaffold only and suture only groups. Both iTenocyte-seeded scaffold and scaffold only treatment groups had improved biomechanical properties when compared to suture only treatment group, however no statistically significant difference was found in comparing the cell seeding scaffold an scaffold only group in terms of biomechanical properties. Immunohistochemistry staining further demonstrated that iTenocytes successfully populated the collagen scaffolds and survived 9 weeks after implantation *in vivo*. Additionally, the repaired tissue of iTenocyte-treated injuries exhibited a more organized structure when compared to tendon defects that were repaired only with suturing or unseeded scaffolds.

**Conclusion:**

We suggest that iTenocyte-seeded DuRepair™ collagen scaffold can be used as potential treatment to regenerate the tendon tissue biomechanically and functionally.

## Graphical Abstract

**Figure:**
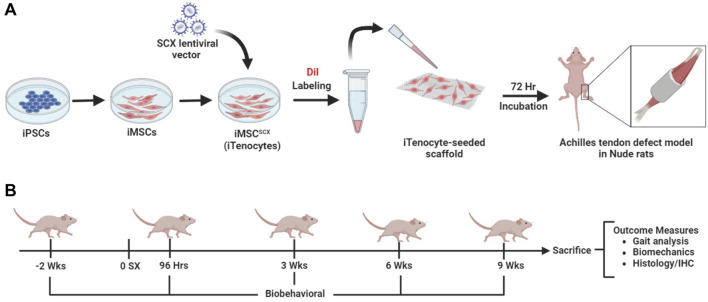
iPSCs were differentiated into iMSCs and transduced with SCX-GFP^+^ lentiviral vector to create iMSC-SCX^GFP+^ **(A)**. The cells were DiI labeled and seeded onto the scaffolds for 72 h and implanted into an Achilles tendon defect model in Nude rats. Rats underwent gait testing before injury, 96 h, 3-, 6- and 9-weeks post injury. After 9 weeks, biomechanical testing and histological analysis was performed to evaluate healing efficacy **(B)**.

## 1 Introduction

Worldwide, about 3 to 5 million musculoskeletal injuries are reported every year due to sports, in which half of these injuries involve tendon and ligament tissue (Giraldo-Vallejo et al., 2023; Gaspar et al., 2015). Acute rupture of the Achilles Tendon is known to be the most common lower extremity tendon injury, with incidences ranging between 7 and 40 per 100,000 persons annually (Sharma and Maffulli, 2005; Wu et al., 2017). Due to increasing life expectancy, the number of such injuries is predicted to further rise in the future, especially in adults participating in sports (Gaspar et al., 2015; Lemme et al., 2018). To treat such defects, nonoperative management strategies and conservative treatments, such as the use of braces, immobilization, or pneumatic walking boots, are currently considered a gold standard (Li and Hua, 2016). Unfortunately, tendon tissue suffers from a poor healing capacity due to minimal blood and oxygen supply, and low metabolic activity, which often results in a long recovery time and a high rate of reinjuries (Xu et al., 2019).

To counteract these limitations, significant developments in stem cell therapies have identified numerous cell types as beneficial to improve tendon healing *in vivo*. Although tendon stem cells have regenerative and self-renewal ability, they are limited in the adult tendon tissue. Furthermore, mesenchymal stem cells (MSCs) have been widely used due to their multipotency, and self-renewability abilities. Despite these positive results, transplanted MSCs are further known to potentially cause immune rejection or tumorigenicity (Lu et al., 2021). Other cell sources such as bone-marrow-derived mesenchymal stem cells and adipose-derived stem cells are more abundant. However, these cells can differentiate into other lineages and ultimately stray from a specific tendon differentiation (Kaneda et al., 2023). In addition, cell expansion *in vitro* can result in phenotypic drift and subsequent functional loss, in addition to low proliferative ability (Jo et al., 2019). Therefore tenocytes differentiated from induced pluripotent stem cells (iPSCs) holds promise due to their unparalleled developmental plasticity, unlimited self-renewal capacity, and the potential scalability for an off-the-shelf cell source application.

Well-established protocols have been developed for the differentiation from pluripotent stem cells to different musculoskeletal cell types including notochordal cells, chondrocytes and osteoblasts by our group and otheres (Wang S. et al., 2013; Kanke et al., 2014; Yamashita et al., 2015; Wu et al., 2021; Sheyn et al., 2016a; Sheyn et al., 2019; Glaeser et al., 2021). Recent studies have successfully differentiated tenocytes using mouse iPSCs and Embryonic Stem Cells (Komura et al., 2020; Nakajima and Ikeya, 2021; Kaji et al., 2021; Yoshimoto et al., 2022). Though, recent work has shown a divergence in developmental processes between mouse and human embryos and subsequent differences in developmental cues on tenogenic differentiation between species (Donderwinkel et al., 2022; Brown et al., 2015; Havis et al., 2014; Havis et al., 2016). Furthermore, other studies have either not reported induction efficiency or showed limited syndetome/tenocyte induction (Komura et al., 2020; Dale et al., 2018). Deriving a homogenous population of tenocytes from iPSCs with high efficiency continues to be a challenge, largely in part to the limited understanding of their developmental origins and differentiation path from multipotential precursors. Therefore in this study, iPSCs have been differentiated into induced MSCs (iMSCs) using a well-established differentiation protocol that have shown no tumorigenic ability (Sheyn et al., 2016a).

To further improve cell-mediated tendon repair strategies, iMSCs can be differentiated towards a tendon-specific lineage by overexpressing Scleraxis (SCX), a transcription factor that is one of the earliest detectable markers for tendon differentiation and adequate embryonic tendon development (Murchison et al., 2007; Huang et al., 2015). iMSCs were transduced with Scleraxis combined with green fluorescent protein (GFP) using a lentivirus vector. Once iMSCs expressed green fluorescence in the nucleus as a surrogate of SCX overexpression, the cells were considered iTenocytes (iMSC^SCX+^). Our previous study has shown that iTenocytes had an upregulation of tendon markers, such as tenomodulin and mohawk homeobox, after 12 days *in vitro* when compared to iMSCs, therefore, making these cells a potential option for tendon regeneration studies (Papalamprou et al., 2023).

To assure an optimal tissue regeneration, cell adhesion is essential upon their implantation *in vivo* (Khalili and Ahmad, 2015). For this purpose, various commercially available biological and synthetical scaffolds, such as high-density hydrogels (Kew et al., 2011) or synthetic polymer-based materials (Cai et al., 2020), were utilized. Although such materials bear the main advantage of having a well-defined 3D structure and allow for a proper integration of cells (Longo et al., 2010), insufficient biomechanical properties to those of native tendon still remain a major drawback (No et al., 2020; Andarawis-Puri et al., 2015; Chen et al., 2009). The Achilles tendon is also known as the strongest, largest, and thickest tendon in the human body because it needs to transmit force by the strongest ankle plantar flexors. Thus, the implanted biomaterials in the Achilles tendon need to be able to mimic and assure local motion and stability to be able to transmit the force (Winnicki et al., 2020). Understanding the biomechanical properties of the tendon is important to create an ideal treatment that should provide a simple operation, reliable fixation, adequate tensile strength, small gap formation, little disturbance to normal Achilles tendon, and little influence on blood supply (Strickland, 1995).

The use of scaffolds for tendon tissue engineering has shown to achieve biological fixation and integrate soft tissue repair after tendon injuries (Vasiliadis and Katakalos, 2020). Scaffolds composed of biological material, such as collagen, have demonstrated biomechanical characteristics and biodegradable properties of the tendon tissue, but their limitations include poor physiological activities such as selective cell adhesion (Baldwin et al., 2018; Hou et al., 2021). One commercially available biomaterial of particular interest is the non-synthetic DuRepair™ Regeneration Matrix, which is comprised of collagen type I and III and offers a highly porous matrix formed by strongly entangled collagen fibers. The collagen material of the scaffold is not only considered biodegradable but also an ideal carrier for cellular attachment and tissue ingrowth (Zerris et al., 2007).

The purpose of this study was to determine the potential of DuRepair™ collagen scaffolds seeded with iTenocytes in promoting the tissue regeneration of a full Achilles tendon defect in a Nude rat model. We therefore hypothesized that iTenocyte-seeded collagen scaffolds will promote both tendon tissue regeneration and biomechanical functionality in Nude rat Achilles tendon defects when compared to suture only repair or unseeded collagen scaffold only.

## 2 Materials and methods

### 2.1 Study design

To optimize cell seeding on DuRepair™ scaffolds, porcine bone marrow-derived MSCs overexpressing luciferase (Luc) reporter gene (pBM-MSC^Luc+^) were seeded at seven different densities (*n = 3*) and analyzed using optical bioluminescence imaging and CellTiter-Glo^®^ cell viability assay.

**FIGURE 1:**
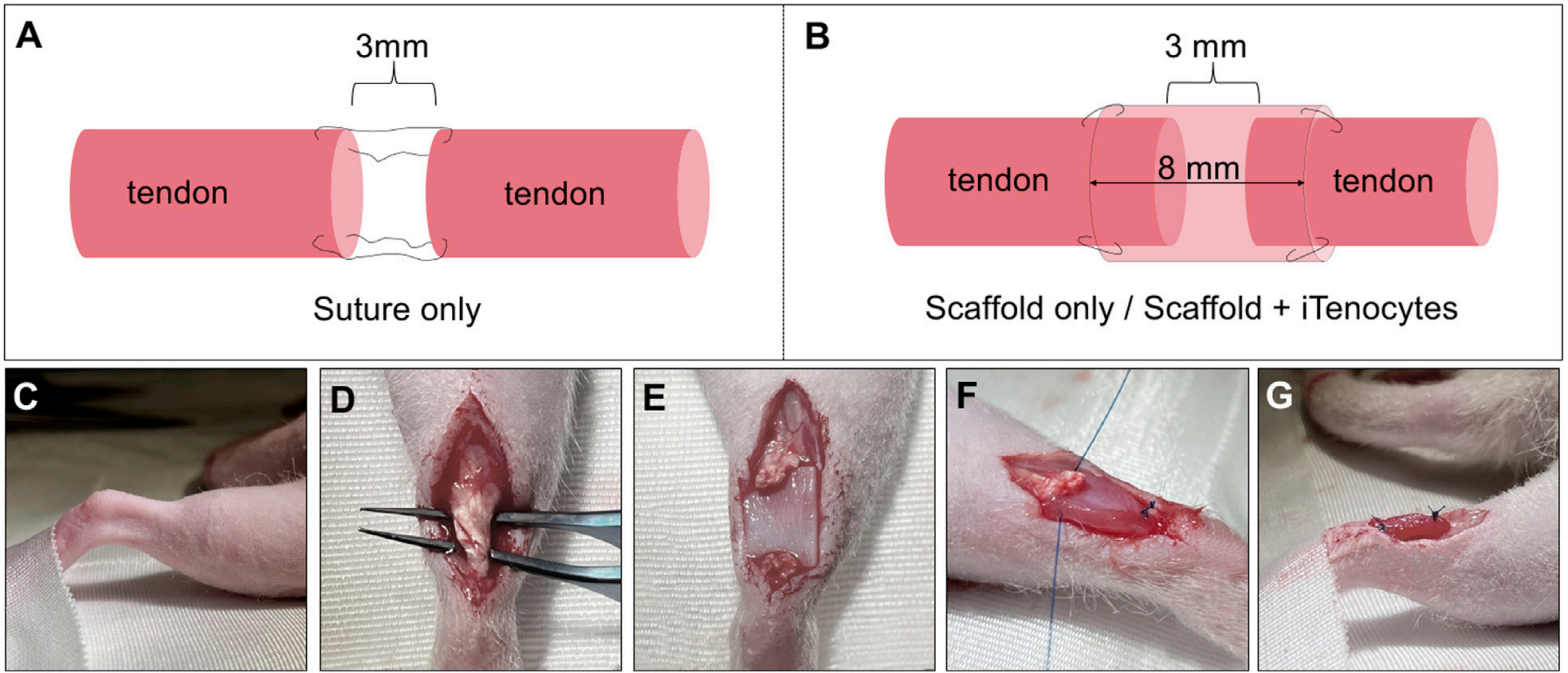
Surgical procedure of Achilles tendon resection and defect treatment. Schematic illustration of defect repair by suture only. After tendon tissue removal, the two ends of tendon were sutured together while maintaining a 3 mm defect **(A)**. Schematic illustration of defect treatment with unseeded or seeded scaffolds. After the creation of a 3 mm defect in Achilles tendon tissue, scaffolds were wrapped around the remaining tendon ends with an overlap of 2.5 mm on each side, leaving a 3 mm gap in the center of the tube-like scaffold structure **(B)**. Images of the *in vivo* procedure **(C–G)**. Right foot of the animal, fixed to the underlaying surface to prevent unwanted moving of the rat **(C)**. After opening skin and muscle layers, a forceps was passed through the tissue underneath the Achilles tendon **(D)**. The Achilles tendon was cut to create a standardized 3 mm tissue defect and the scaffold was placed underneath the defect, with the cell-seeded side of seeded scaffolds towards the tendon **(E)**. Both ends of the scaffolds were sutured to the ends of the tendon and maintained the 3 mm standardized gap, resulting in a tube-like structure of the scaffold, wrapping it around the defect **(F, G)**.

Following *in vitro* seeding optimization, 5 × 10^5^ iTenocytes per 40 µL of media were seeded onto DuRepair™ collagen scaffolds (5 mm × 8 mm × 1 mm) and cultured for 72 h at 37°C and 5% CO_2_ before implantation. Subsequently, the seeded scaffolds were used to repair 3 mm Achilles tendon defects in immunocompromised Nude rats *in vivo* (*n = 12*). Tendon defects treated with unseeded scaffolds (*n = 12*) or sutured without adding scaffold to the defect (*n = 12*) served as two control groups (Figures 1A, B). Gait testing was done within 2 weeks period before surgery (baseline) as well as 96 h, 3-, 6- and 9 weeks after injury and implantation. Following the animals’ sacrifice at week 9, excised tendon tissues were biomechanically tested or processed for histological and immunohistochemical analyses.

### 2.2 Cell culture

Human iPSCs were obtained from the Cedars Sinai iPSC core facility and subsequently differentiated towards mesenchymal lineage (iMSCs) as we have previously reported (Sheyn et al., 2016a). The iMSCs were transduced with Scleraxis and GFP using a lentiviral vector as reported (Papalamprou et al., 2023; Yu et al., 2024). Briefly, HEK293T/17 cells (ATCC, Manassas, VA) were seeded at a density of 5,300 cells/cm^2^ in Eagle’s Minimum Essential Medium (EMEM, #30-2003, ATCC) containing 10% fetal bovine serum (FBS, #100-106, Gemini Bio, West Sacramento, CA) and 1% antibiotic-antimycotic solution (AAS, #15240096, ThermoFisher, Waltham, MA). After 24 h, lentivirus was produced by transfecting the HEK293T/17 cells with 7.5 µg of Luciferase plasmid or 7.5 µg of SCX-GFP plasmid (OriGene, Maryland, United States) and two packaging plasmids (pCMV-dR8.2, 6.75 µg; pCMV-VSV-G, 0.75 µg; all three plasmids were gifted by the Simon Knott laboratory at Cedars-Sinai Medical Center). The transfections were conducted using the BioT method with a 1.5:1 ratio of BioT (μL) to DNA (μg). The media containing the Luciferase or SCX-GFP lentiviral vectors was harvested after two and 3 days of transfection and filtered using a 45 µm filter. The iMSCs or pBM-MSCs were seeded at a density of 4,000 cells/cm^2^ in complete medium. They were transduced with SCX-GFP or Luciferase viral vectors as well as lenti-packing medium (complete medium, 10% heat-inactivated FBS, 1% L-glutamine) on the following day. Two days after viral vector was added, cells were washed with PBS and complete medium was added to the cells. The successful transduction of iMSCs with SCX was validated with green fluorescence in the nucleus (Papalamprou et al., 2023). The transduced iMSC-SCX^GFP+^ (iTenocytes) and pBM-MSC^Luc+^ were grown until 90% confluency and used for experimentation. The growth medium that was utilized for pBM-MSCs was high glucose DMEM that contained 10% FBS, 1% AAs, and 1% L glutamine.

### 2.3 Scaffold preparation and cell seeding

As carrier material for this study, acellular DuRepair™ Regeneration Matrix (MedTronic, MN, United States) was used. The material is comprised of type I and type III collagen and has a pore size of 10–20 µm (Prickett and Wise, 2013; McCall et al., 2008). For *ex vivo* biomechanical testing, the scaffold was cut into samples of 5 mm^3^ × 15 mm^3^ × 1 mm^3^ (*n = 6*). For *in vivo* implantation, scaffolds of 5 mm^3^ × 8 mm^3^ × 1 mm^3^ dimensions were generated. Scaffolds were placed into a glass Petri dish and placed under UV for 15 min. For determination of an optimal cell seeding density on collagen scaffolds, 10,000, 25,000, 50,000, 125,000, 250,000, 500,000, and 1,000,000 pBM-MSC^Luc+^ were seeded onto scaffolds of the same dimensions. To do so, 48-well plates were coated with Poly-Hema (2-hydroxyethyl methacrylate) to prevent cellular attachment outside of the scaffold. Subsequently, the scaffold samples (*n = 3*) were placed inside the well plate and the desired cellular concentration was slowly and homogeneously pipetted onto the scaffold in a total volume of 40 µL of Gibco™ low glucose Dulbecco’s Modified Eagle Medium containing 10% fetal bovine serum (FBS), 1% Antibiotic and Antimycotic (AAs), and 1% L-Glutamine. After seeding, scaffolds were incubated at 37°C, 5% CO_2_ for 4 h to allow cell attachment. Subsequently 500 µL of medium were slowly added into each well and all samples were imaged using bioluminescence.

### 2.4 Cell viability assessment

To determine the attachment and retention of cells on the scaffolds, the seeded biomaterials were repetitively analyzed by means of bioluminescence imaging and CellTiter-Glo viability assay (Promega, Madison, WI). Bioluminescence imaging was first used to quantify cellular viability and retention at 4 consecutive culture days by deploying luciferase reporter gene expressing cells as previously reported (Sheyn et al., 2011; Kimelman et al., 2013; Sheyn et al., 2016b; Kremen et al., 2019). In detail, 2.5 µL of luciferin (15.75 mg/mL) were added to each well containing one seeded scaffold and 500 µL of medium. The well plates were carefully shaken for 5 s to assure a homogeneous distribution of the luciferin, incubated for 2 min, and imaged using IVIS Spectrum imaging system (Perkin Elmer, Valencia, CA). Quantification was done with the Living Imaging software (version 4.7.3; Perkin Elmer).

To verify cell viability CellTiter-Glo™ Luminescence assay was used to assess the end-point cell viability via quantification of adenosine triphosphate (ATP) release as an indicator of metabolic active cells. Briefly, seeded scaffolds were removed from their culture conditions and each sample transferred into a 1.5 mL tube containing 125 µL CellTiter-Glo™ reagent diluted in 100 µL PBS and homogenized to induce cell lysis. The samples were then centrifuged at 13,000 RPM for 3 min. Lastly, 50 µL aliquots of the supernatant were placed in a well of a 96-well plate (n = 3). As a control group, CellTiter-Glo™ reagent and PBS without a scaffold was used. To stabilize the luminescence signal, plates were incubated at room temperature for 25 min before being measured in a spectrophotometer at 426 nm.

### 2.5 Achilles tendon defect survival surgery

Prior to conducting the surgical procedure, appropriate Institutional Animal Care and Use Committee (IACUC) approval was obtained. For the *in vivo* surgical part, a total of 35 females athymic Nude rats (Strain: NIH‐Foxn1rnu, Substrain: Ctr; Charles River) were randomly assigned into one of three cohorts according to previously described treatment groups including suture only (*n = 11*), scaffold only (*n = 12*), or iTenocyte-seeded scaffold (*n = 12*). Based on power analysis, variability of control group (suture only) was lower; therefore, we needed a smaller sample size (Kaneda et al., 2023). The Nude rats were 18 weeks old, and their weight was 227.5 ± 19.6 grams during the day of surgery. Female rats were chosen because they are less aggressive and can be easily handled for our staff. On the other hand, no significant differences were found between females and male animals in terms tendon mechanical properties and function (Sarver et al., 2017).

First, animals were anesthetized with isoflurane inhalation (4%). Once deep anesthesia was confirmed, the hind limb was shaved, and the surgical area sterilized with anti-septic Betadine solution (Figure 1C). A heating pad was placed underneath the animals to prevent hypothermia. Once the right hind limb was extended and fixed to the underlying surface of the surgical table, a 1.5–2 cm wide incision, superficial to the Achilles tendon, was made using a scalpel blade (#15). Using forceps and blunt dissection with scissors, the Achilles tendon was exposed and cleared from surrounding adipose and muscle tissue (Figure 1D). Once a clear exposure was achieved, 3 mm of the Achilles tendons’ midpoint region was excised. For animals that were treated with suture only, a 6‐0 Prolene suture (8706H, Ethicon^®^, Johnson and Johnson; Raritan) was used to secure the two free ends of the cut Achilles tendon while, at the same time, maintaining a gap of 3 mm between the two free ends of the tendon tissue via the Modified Kessler suturing technique. For both other groups (scaffold only and iTenocyte-seeded scaffolds), the seeded and unseeded biomaterials were placed underneath and wrapped around the tendon. Noteworthy, the cell-seeded side of scaffolds containing iTenocytes was facing upwards (Figure 1E). Subsequently, a 6‐0 Prolene suture was used to secure one side of the scaffold to the free end of the tendon with one suture. While maintaining the gap of 3 mm in the mid-section of the scaffolds, the second end of the biomaterial was fixed to the free tendon tissue with one suture as well. Following this, a second suture was used on both ends to fix the scaffolds after wrapping them around the tendon tissue, creating an enclosed, tube-like structure (Figures 1F, G). This structure allowed the cell-seeded area to be on the inside of the material. The incision was then closed via simple interrupt suturing method by means of an interrupted 3‐0 Ethilon sutures (1669H, Ethicon^®^, Johnson and Johnson; Raritan).

Following the survival surgery, rats were placed in solitary recovery cages with gel diet on the cage floor. Antibiotics were given subcutaneously on the day of and after surgery. Buprenorphine was administered at 0.1 mg/kg subcutaneously for 2 consecutive days post-surgery according to the IACUC protocol. All rats were euthanized 9 weeks after surgery using isoflurane (33%) overdose in a desiccator jar followed by physical method to assure death.

### 2.6 Achilles tendon defect functional recovery testing

To assess the tendon healing *in vivo*, a functional gait analysis was performed. A modified version of the Achilles Functional Index, established by Murell et al., was used as previously described (Kaneda et al., 2023; Webb et al., 2013; Murrell et al., 1992). Briefly, after non-toxic ink (red and blue) was applied to front and back paws of the rats, the animals were placed at one end of a white paper-lined corridor (∼10 cm × 48 cm). Subsequently, the rats were placed between two barriers to walk through the corridor. Paw prints of all animals were obtained before surgery (baseline), as well as 96 h, 3-, 6-, and 9 weeks post-surgery.

**FIGURE 2:**
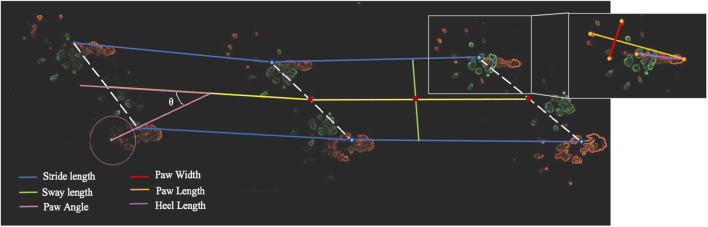
Functional recovery gait test of Achilles tendon defects in Nude rats. Gait testing sheet with hind paws in orange and fore paws in green. Stride length (blue), sway length (green), paw width (red), paw length (orange), paw angle (pink) and heel length (purple) were measured using the hind paws.

Collected paw prints were scanned to assess stride length, paw width, paw angle, heel length, and sway width of hind limbs (Figure 2) using Fiji ImageJ analysis software (version 1.0). Stride length was defined as the distance between the most proximal points of the thenar pad between two adjacent steps made by the same foot. Stride width was defined as the width at midpoint, made perpendicular from the midline between two opposite strides. The degree of external rotation of the hind paw was measured relative to the long axis of the animal’s direction of locomotion. Paw angle was defined by using the most proximal points of the second through fourth metatarsals to create a circle. After assessing the midpoint of the circle, a line was extended from the midpoint through the thenar pad to the midline. The angle created by the midline and thenal pad line was then measured. Heel length and paw width were defined as the most distal point of the thenar pad and most proximal point of the heel as seen on the print and measuring the length between most distal points of the first and fifth distal phalanges, respectively.

### 2.7 Biomechanical material testing

**FIGURE 3:**
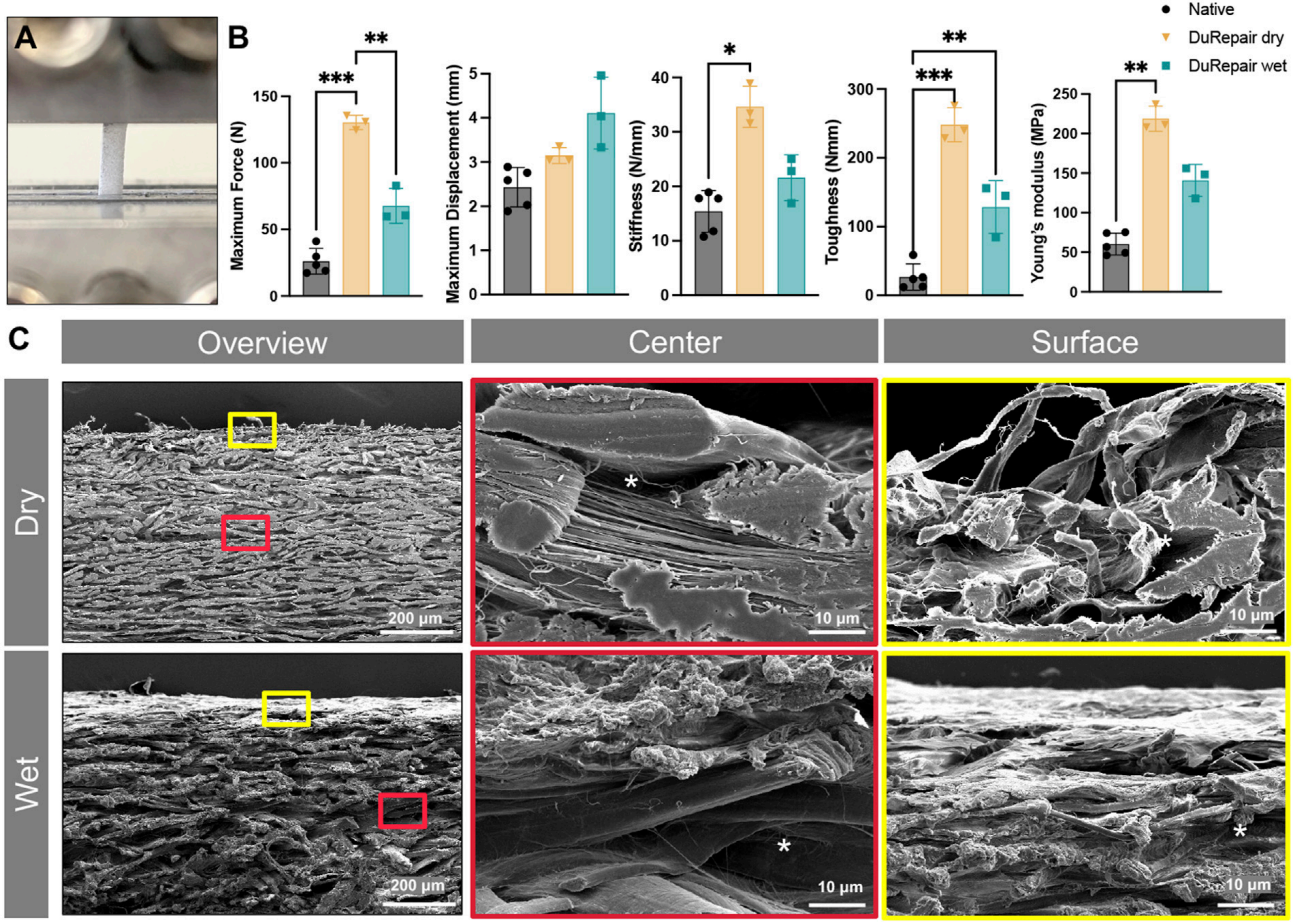
Material characterization DuRepair™ collagen scaffolds. Representative image of a dry DuRepair™ scaffold fixed with two mechanical clamps for biomechanical testing **(A)** Biomechanical outcome measures (maximum force, maximum displacement, stiffness, toughness, and young’s modulus) of native tendon tissue (●), DuRepair™ dry (▼), DuRepair™ wet (■). **(B)** n = 3 *p < 0.05 **p < 0.01 ***p < 0.001. Representative scanning electron microscopic cross section images of dry and wet DuRepair™ collagen scaffolds. Surface (red outlines) and center (yellow outlines) zones represent higher magnifications of wet and dry overview images **(C)**.

To mimic the intended use *in vivo*, scaffolds (5 mm^3^ × 15 mm^3^ × 1 mm^3^; *n = 6*) were wrapped around to create a cylindrical shape equivalent to how the scaffold appears *in vivo* after wrapping it around the tendon tissue. Half the scaffolds (n = 3) were tested dry whereas the other half (*n = 3*) was placed in a 15 mL test tube filled with PBS for 5 min before testing to mimic the wet conditions when implanted *in vivo.* Both ends of the scaffolds were clamped between serrated metal plates and tensile testing of wet and dry scaffold was performed using the same loading protocol described below for the *ex vivo* tendons (Figure 3A).

For the *ex vivo* part of the study, 26 rats underwent biomechanical testing post sacrifice (*n = 8* for suture only, *n = 9* for scaffold only, and *n = 9* for iTenocyte-seeded scaffolds). As mentioned previously, a smaller sample size was needed in suture only group because variability of control group was lower on power analysis (Kaneda et al., 2023). Native Achilles tendon controls were harvested from the contralateral limbs of all rats from of all three treatment groups. To harvest the tendon for uniaxial testing, the Achilles was grossly dissected to its point of origin at the gastrocnemius while leaving the insertion point in the calcaneus intact. The surrounding musculature was carefully removed via blunt dissection to expose the tendon. By means of a #15 scalpel blade, sheath of tissue adherent to the tendon was removed in a distal to proximal sweeping motion. The distance between the calcaneus insertion and the Achilles‐gastrocnemius junction as well as the diameter and thickness (frontal and sagittal plane) at the mid‐substance were measured using a caliper to determine the gauge length and calculate the cross‐sectional area using an ellipse formula. The biomechanical testing was performed as previously reported (Kaneda et al., 2023). Briefly, the proximal Achilles tendon tissue was held between serrated metal plates via bolts that were torqued to 1 Nm with a manual torque wrench. Distally, the foot was rigidly fixed to custom-built jig that was attached to a metal plate via an 1/8 in. zinc‐plated wire rope clamp. The jig was secured to the frame of the hydraulic mechanical testing system (370.02 Bionix, MTS Systems Corp.). Specimens were pre-loaded to 1 N, followed by load to failure at a rate of 0.15 mm/s, indicated by a 30% drop in load. Both displacement and tensile force were continually recorded. Toe region displacement, ultimate load, displacement at failure, and stiffness were calculated from the resultant force‐displacement curve. Maximum strain, toughness, maximum stress (maximum stress that a material can withstand before it breaks or weakens), and Young’s Modulus (slope of the linear part of the stress-strain curve) were calculated from the stress-strain curve.

### 2.8 Histology and immunofluorescence

After rats were sacrificed at 9 weeks post-surgery, Achilles tendon tissues were harvested for histology and immunofluorescence from 9 rats (n = 3 per group) as previously reported (Kaneda et al., 2023). Briefly, Achilles tendons and parts of the soleus gastrocnemius tissue were explanted, cleared from muscle tissue, fixed in 4% formaldehyde solution, passed through a graded ethanol solution series, and finally embedded in paraffin. Subsequently, 5 µm-thick sections were cut out of the paraffin blocks and used for Hematoxylin and Eosin (H&E) staining or for Masson’s trichrome to evaluate tissue morphological features and the matrix formation of the healing tendons.

For immunofluorescent staining, tissues were deparaffinized, and antigens retrieved by incubation in Proteinase K (Agilent) for 20 min at room temperature. Nonspecific antigens were blocked through the application of Normal Donkey Serum (Jackson ImmunoResearch). After primary antibodies were applied according to Supplementary Table S1, slides were incubated at 4°C overnight and washed using PBS (10x). Subsequently, the slides were incubated with secondary antibodies for 1 h at room temperature. No primary antibody-treated samples were used as negative controls to ensure specificity of staining. Finally, all slides were stained with DAPI for 5 min under the absence of light. Revolve microscope, model RVL-100-B2, via ECHO-pro software was used to capture fluorescent images.

Collagen fiber orientation differs between native and injured tendon, changing from regular to random, respectively (Sivaguru et al., 2010). Nuclei orientation has been used in previous studies as a proxy to assess collagen fiber linearity to better understand tendon tissue health and this inspiration was utilized in our assessment of collagen fiber linearity (Papalamprou et al., 2023). H&E-stained slides of the samples were scanned and processed using QuPath software (v0.5.1). The eosin channel was hidden for each slide. At 20x magnification, five non-overlapping sections (400 μm × 800 µm) of the tendon tissue were chosen at random for each slide. The view of the section was rotated so that the nuclei were at a baseline of being as close to a 0-degree angle as possible. Each of the five sections of tendon tissue per slide was uploaded to ImageJ2 software (v2.14.0). The contrast of each image was increased until the background hematoxylin staining was lightened, and the nuclei were darkened. The image was converted to binary and skeletonized. The Feret’s Diameter measurement package was used for each image, and particles larger than 10 pixels were measured to remove background noise. After measurements were obtained, the Feret Angle was analyzed for each nucleus. Angles that were greater than 90° were subtracted by 180 to move data points from quadrant 2 to quadrant 4 of the coordinate plane, and then the absolute value of each angle was taken to assess the displacement of each angle from 0. Data points for each of the four groups were graphed using a rose plot, and statistical analysis was conducted using the Kolmogorov-Smirnov Test to assess the variability of each of the groups.

### 2.9 Scanning electron microscopy

To visualize surface topography and cellular attachment of DuRepair™ samples 3 days after seeding, samples were processed for scanning electron microscopic (SEM) analyses. First, the samples were dehydrated in an ascending series of denatured ethanol (70%, 80% and 90% for 10 min. each). Finally, the samples were incubated in pure ethanol (96% and 100% for 10 min each). Dehydration was completed by incubation in a freshly prepared mixture (1:1) of 100% ethanol and hexamethyldisilazane (HMDS, Carl Roth GmbH + Co. KG, Karlsruhe, Germany), followed by two further incubations of pure HMDS (10 min each) and an overnight incubation in HMDS. Uncoated scaffolds were cut into smaller pieces to allow visualization of both the surface and cross section. All specimens were transferred to conductive carbon adhesive tabs on standard SEM pin stubs (Micro to Nano, Haarlem, Netherlands), sputtered with carbon (SCD 030, Balzers Union, Liechtenstein), and additionally with gold (SCD 005, Balzers Union) for 3 × 60 s to minimize charging of the highly porous specimens. SEM analysis was performed on a FEI XL 30 ESEM FEG SEM machine (Hilsboro, Oregon, United States) in secondary electron mode, with an accelerating voltage of 5 kV and a working distance of 10 mm.

### 2.10 Statistical analysis

All statistical analyses were performed using Prism 8 (GraphPad) with *p* < 0.05 noted as statistically significant. Normality tests, such as data points over two standard deviations from the average, were excluded from analysis. For gait analysis, 2‐way analysis of variance (ANOVA) or mixed effects analysis, were performed for each dependent measure separately, using mean values with grouping of experimental groups. For multiple comparisons, appropriate *post hoc* tests were used. For biomechanical testing, an ordinary one‐way ANOVA was performed. In the figures, average (±SD) values are shown. These results demonstrate that DuRepairTM collagen scaffold has biomechanical properties similar to the native tendon tissue of Nude rats, making this scaffold a promising candidate for tendon tissue engineering purposes.

## 3 Results

### 3.1 Material characterization of DuRepair™ collagen scaffold

To evaluate the suitability of DuRepair™ scaffold for tendon regeneration, we first analyzed its biomechanical parameters and compared to native rat Achilles tendon tissue (Figures 3A, B). Since the *in vivo* implantation of the biomaterial would be in a wet state following cell culturing, we also tested DuRepair™ samples in a wet state. Of interest, biomechanical analyses of wet scaffolds revealed no significant differences in maximum force (N), maximum displacement (mm), stiffness (N/mm, and young’s modulus (MPa), when compared to native rat tendon tissue, making this collagen scaffold a promising candidate for tendon and ligament tissue engineering purposes.

In a second set of experiments, we analyzed the surface topography of the scaffold when it was dry and wet (Figure 3C) biomaterial by means of SEM. We could demonstrate that both dry and wet samples revealed a highly dense structure with only small pores (asterisks) at the border (yellow frames) and center (red frames) zones. Although the saline-soaked scaffolds visibly exhibit a swelling, the overall structure remained comparable. These results demonstrate that DuRepair™ collagen scaffold has biomechanical properties similar to the native tendon tissue of Nude rats, making this scaffold a promising candidate for tendon tissue engineering purposes.

### 3.2 Evaluation of cell viability and attachment when seeded on collagen scaffold *in vitro*

**FIGURE 4:**
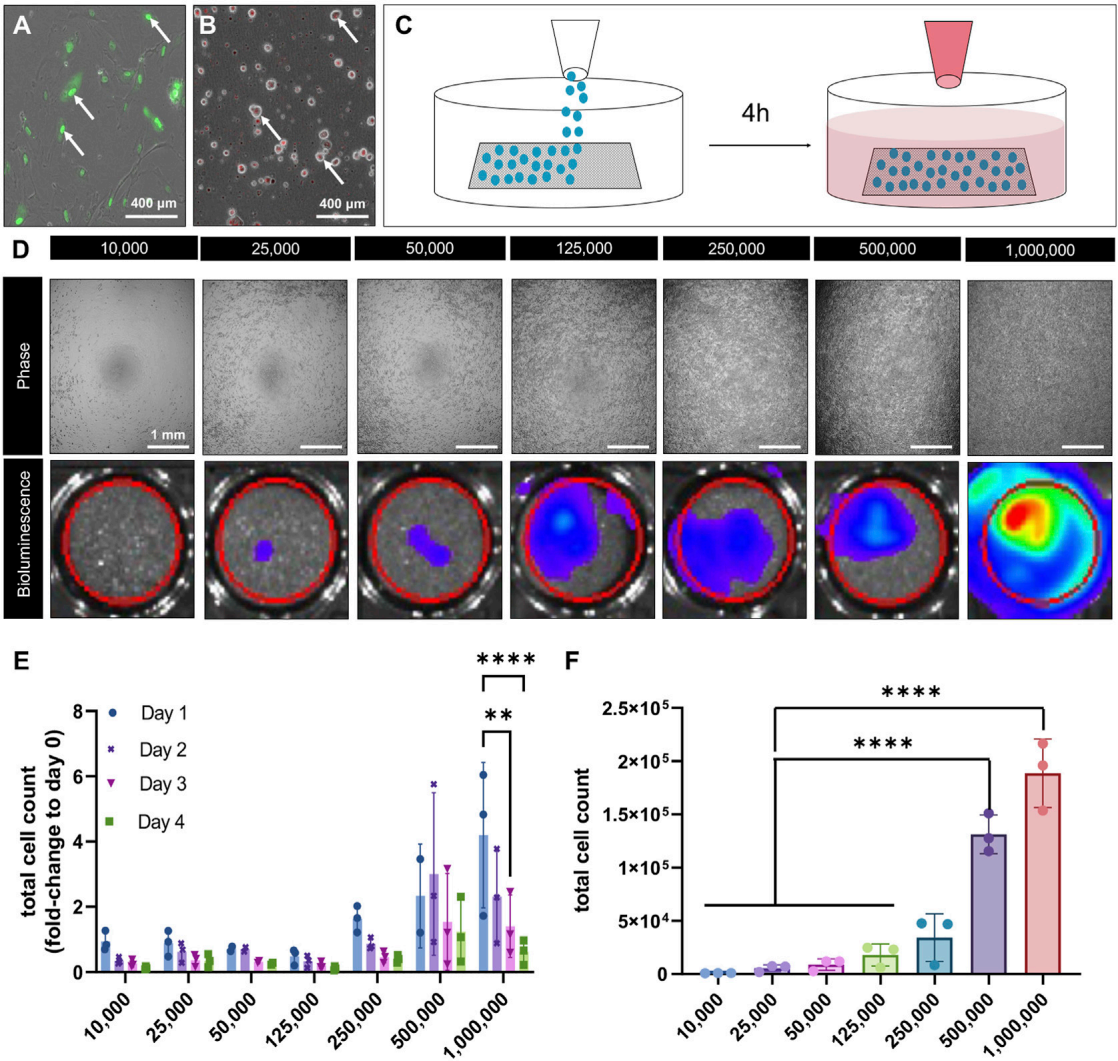
Viability of pBM-MSCLuc+ seeded collagen scaffolds *in vitro*. Representative confocal microscopy image of iTenocytes in culture, in which the nucleus is expressing SCX-GFP fluorescence **(A)**. Representative image of DiI-labeled pBM-MSC-Luc+ in culture **(B)**. Schematic illustration of the cellular seeding process. pBM-MSC-Luc cells were homogeneously seeded on top of collagen scaffolds and incubated for 4 h before medium was added to assure proper cellular attachment **(C)**. Light microscopic images of cell-seeded scaffolds on non-adherent Poly(2-hydroxyethyl methacrylate)-coated wells (top) and bioluminescence images showing increasing bioluminescent signals of scaffolds seeded (bottom) ranging from 10,000, to 1,000,000 cells **(D)**. Total cell count of seeded scaffolds from days 1 (●), day 2 (**✖**), day 3 (▼), and day 4 (■) given as fold-change to day 0 (right after seeding) **(E)**. Total cells count of attached cells after 4 days of seeding via cell viability assay **(F)**. **p < 0.01, ****p < 0.0001, n = 3.

The iTenocytes used in this study are expressing SCX tagged with GFP (Figure 4A). However GFP has relatively long half life and to monitor cell survival and retention to the scaffold other Luciferase transduced cells were used. To determine the optimal cell seeding density as well as the optimal time for implantation after seeding, we analyzed the viability of DiI-labeled Luciferase reporter gene-transduced cells (Figure 4B) following their seeding onto 5 × 8 mm^2^ scaffolds (Figure 4C).

After seeding 10,000, 25,000, 50,000, 125,000, 250,000, 500,000, or 1,000,000 cells in 2D cell culture wells and performing IVIS bioluminescence imaging, a standard curve was created (Figure 4D). Seeded scaffolds underwent IVIS bioluminescence imaging from day 0 to day 4, and total cell counts were calculated using the standard curve and were then normalized to day 0. We found that seeding 1,000,000 cells led to a significant cell loss over the 4-day time period (Figure 4E). Moreover, scaffolds seeded with 1,000,000 cells revealed a comparable viability after 48 h and 72 h to those scaffolds seeded with 500,000 cells. To confirm these finding, we conducted an end-point analysis of the seeded scaffolds on day 4 using CellTiter-Glo^®^ luminescent cell viability assay (Figure 4F). In line with our IVIS bioluminescence imaging, this test revealed that the viability drastically increased from 250,000 to 500,000 and 1,000,000 cells. However, no significant difference in viability could be detected between 500,000 and 1,000,000 cells (Figure 4F). Accordingly, scaffolds were seeded with 500,000 pBM-MSC^Luc+^ and cultured for 3 days before being implanted into critical size rat tendon defects.

### 3.3 Cellular attachment and surface topography of cell-seeded collagen scaffold

**FIGURE 5:**
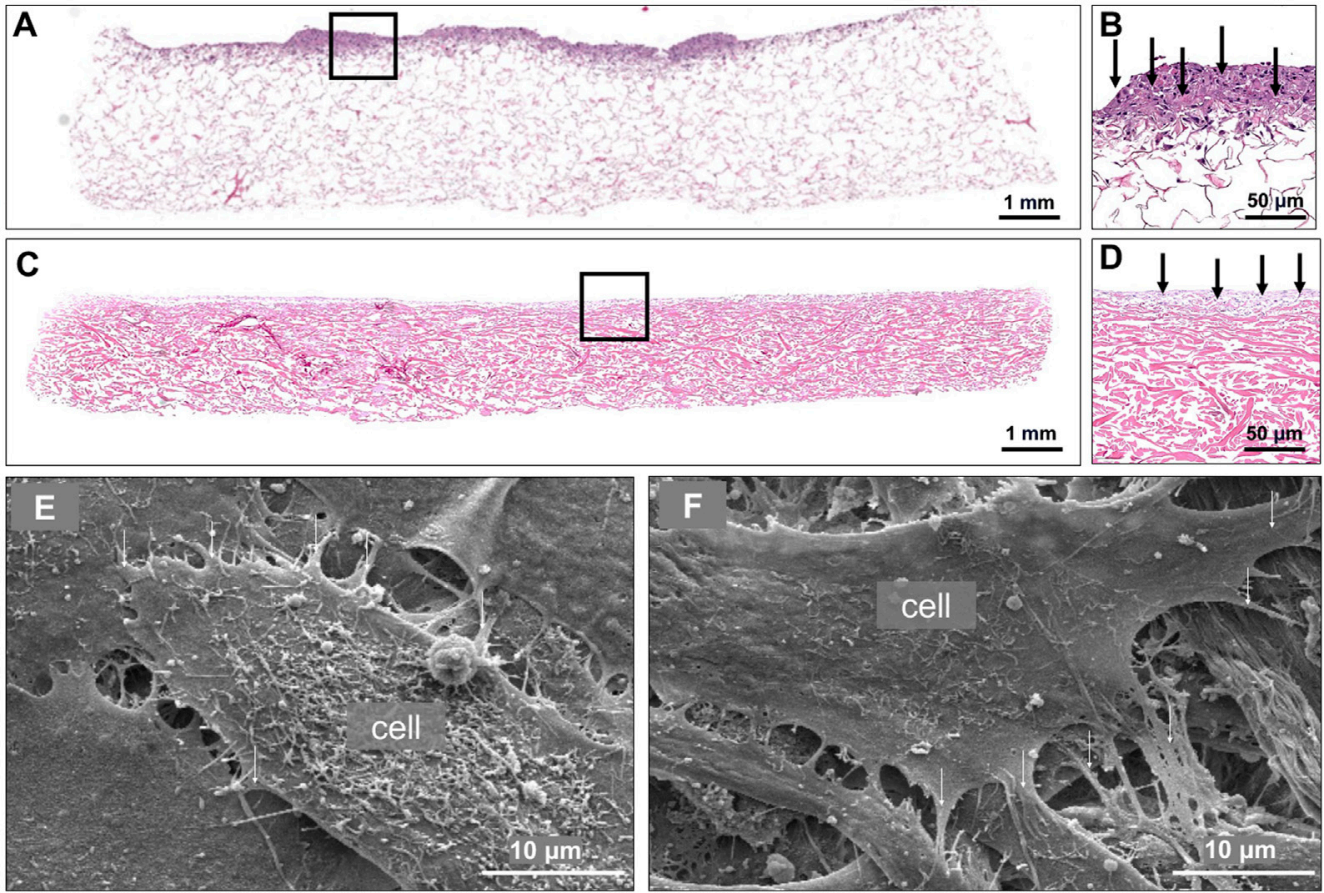
Cellular attachment and surface topography of cell-seeded collagen scaffolds. Representative H&E-stained section of collagen scaffold 4 h after seeding with 500,000 pBM-MSC-Luc+ **(A)**. Higher magnification of A, showing cellular presence in exclusively the superficial zone of the scaffold **(B)**. Representative H&E-stained section of a collagen scaffold 3 days after seeding with 500,000 pBM-MSCLuc+ **(C)**. Higher magnification of C, showing cellular presence in exclusively the superficial zone of the scaffold **(D)**. Two representative scanning electron microscopic images of a collagen scaffold seeded with 500,000 pBM-MSCLuc+. Arrows point to the attached cells **(E, F)**.

Following the cell viability analyses and scaffold seeding optimization *in vitro*, samples were prepared for histological assessment and SEM. H&E-stained scaffolds, seeded with 500,000 DiI-labeled pBM-MSC^Luc+^ 4 h (Figures 5A, B) and 3 days (Figures 5C, D) after seeding, revealed a relatively shallow seeding depth, however not in a monolayer. Furthermore, when we looked at the surface under high magnification using SEM (Figures 5E, F), proper cellular attachment to the scaffold and between the cells was visualized.

### 3.4 Functional recovery of achilles tendon defect repaired with iTenocytes-seeded collagen scaffold *in vivo*

**FIGURE 6:**
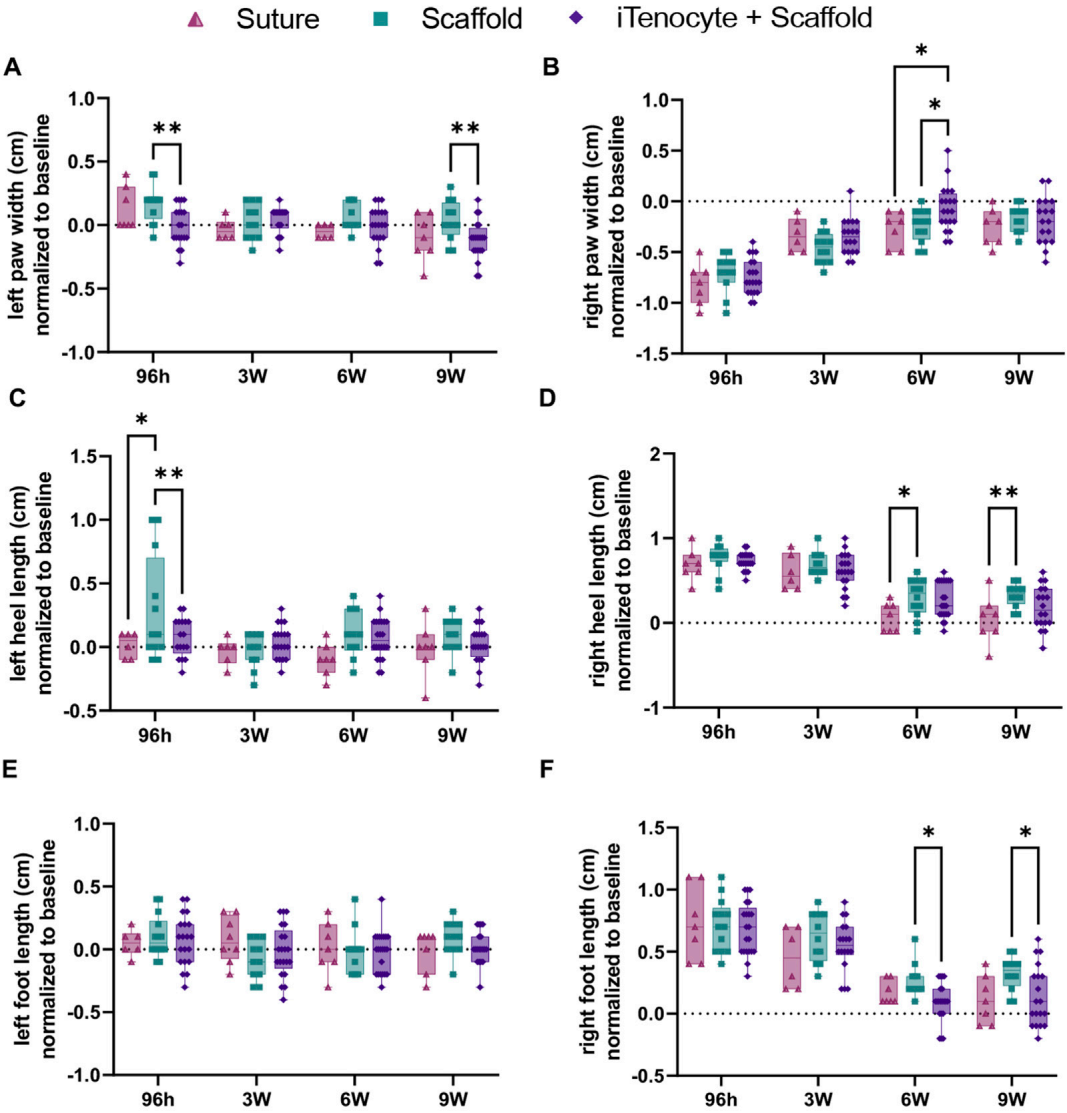
Functional recovery of Achilles tendon defects treated with iTenocyte-seeded collagen scaffolds *in vivo*. Paw widths of left **(A)** and right **(B)** paws. Heel lengths of left **(C)** and right **(D)** paws. Foot lengths of left **(E)** and right **(F)** paws 96 h, 3-,6- and 9- weeks after injury and treatment of suture (▲), scaffold (■), and iTenocyte-seeded scaffold (**♦**). Baseline is represented as the dotted line. *p < 0.05, **p < 0.01, ***p < 0.001, ****p < 0.0001. Suture only n = 11; scaffold only n = 12, scaffold with iTenocytes n = 12.

After surgical removal of 3 mm Achilles tendon tissue, the defects were repaired by either suture only, scaffold only, or iTenocyte-seeded scaffold (Figure 1). To assess the functional healing of the tendon gait analysis was performed 2 weeks prior to surgery (baseline, represented as the dotted line) as well as 96 h, 3 weeks, 6 weeks, and 9 weeks post injury. No significant differences were found between left or right stride lengths of all treatment groups (Supplementary Figures S1A, B). iTenocyte-seeded scaffold-treated animals 96 h after injury, revealed a left (uninjured) paw width similar to the baseline and when compared to scaffold-treated animals. The iTenocyte-seeded scaffolds showed significantly less changes 96 h and 9 weeks after injury when compared to unseeded scaffolds group. On the other hand, there was no significant difference in left paw width between iTenocyte-seeded scaffold and suture only group (Figure 6A). Further analysis of the right (injured) foot revealed a highly significant change of paw width in all three groups when compared to baseline both 96 h and 3 weeks after injury. Of interest, from week 6 postinjury onward, animals treated with iTenocyte-seeded scaffolds revealed a paw width comparable to baseline, indicating a better and faster recovery, whereas other treatment groups showed similar results only at week 9 (Figure 6B).

Additionally, both iTenocyte-seeded scaffold group and suture only-treated animals revealed a left heel length comparable to baseline compared to scaffold only group (Figure 6C). In line with its paw width, the right heel length in all treatment groups was significantly larger from baseline after 96 h and 3 weeks post injury. No significant differences were observed for these timepoints between the three animal groups. The suture only group had a right heel length similar to baseline while the both the scaffold only and iTenocyte-seeded scaffold had slightly larger heel lengths in week 6 and 9 post injury (Figure 6D). Analysis of the left foot lengths did not reveal any significant differences (Figure 6E). However, the right feet of all animals revealed a continuous healing progression over time. Starting from week 6, the iTenocyte-seeded scaffold-treated rats showed a right foot length comparable to the baseline measurement, whereas when compared to unseeded scaffolds a significant difference was observed. There was no significant difference in right foot length between the iTenocyte-seeded scaffold and suture only groups on week 6 and 9 post injury (Figure 6F).

### 3.5 Biomechanical characterization of repaired defects

**FIGURE 7:**
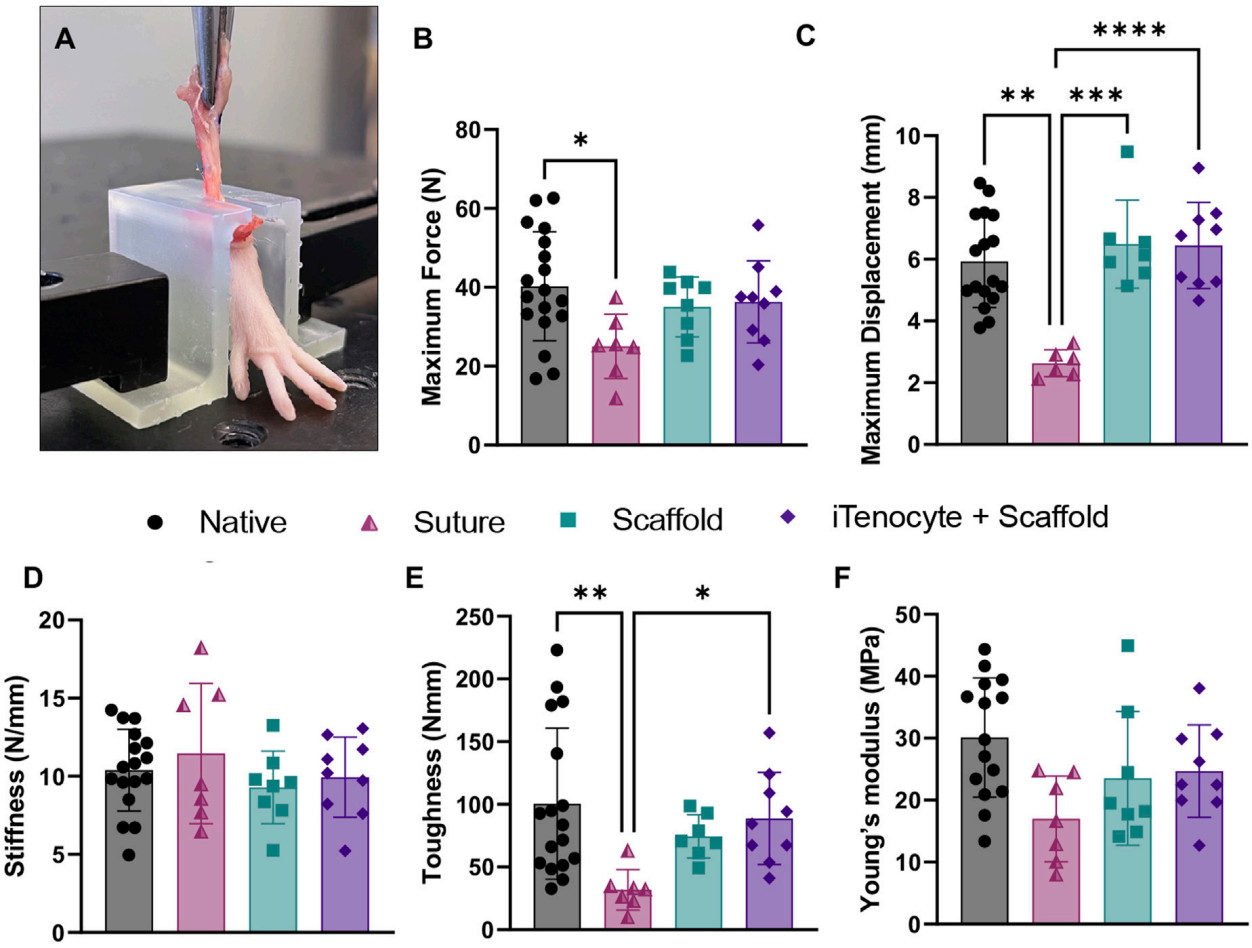
Biomechanical characterization of repaired defects. Representative image of a rat foot mechanically fixed onto MTS machine, while continuous mechanical stretching is applied by upward movement onto the treated Achilles tendon tissue **(A)**. Biomechanical outcome measures maximum force **(B)**, maximum displacement **(C)**, stiffness **(D)**, toughness **(E)**, and young’s modulus **(F)** of native tendon tissue (●) suture (▲), scaffold (■), and iTenocyte-seeded scaffold (♦). *p < 0.05, **p < 0.01, ***p < 0.001, ****p < 0.0001.

Nine weeks post-injury, all animals were sacrificed, and Achilles tendon tissues were explanted to test biomechanical properties using an MTS machine as previously described (Kaneda et al., 2023) (Figure 7A). Suture only treated animals revealed a significantly diminished maximum force when compared to native tissue, while scaffold only and iTenocyte-seeded scaffold groups had comparable maximum forces to native tendon tissue (Figure 7B). iTenocyte-seeded scaffold-treated tendons revealed a maximum displacement comparable to that of native tissue while unseeded scaffolds resulted in a maximum displacement comparable to that of suture only-treated animals, which presented with a significantly lower value than native tissue (Figure 7C). The iTenocyte seeded-scaffold and scaffold only groups had no significant differences in biomechanical properties, when compared to each other. Only in toughness the Scaffold+iTenocyte group was superior to suture only, whereas scaffold only was not.

Defects treated with suture only resulted in a significantly reduced toughness when compared to native rat Achilles tendon tissue. On the other hand, defects treated with unseeded scaffolds or iTenocyte-seeded scaffolds revealed a toughness comparable to that of native tissue (Figure 7E). No significant changes were detected for stiffness (Figure 7D) and young’s modulus (Figure 7F) for all treatment groups.

### 3.6 Histology and immunohistochemistry of achilles tendon defects *in vivo*

**FIGURE 8:**
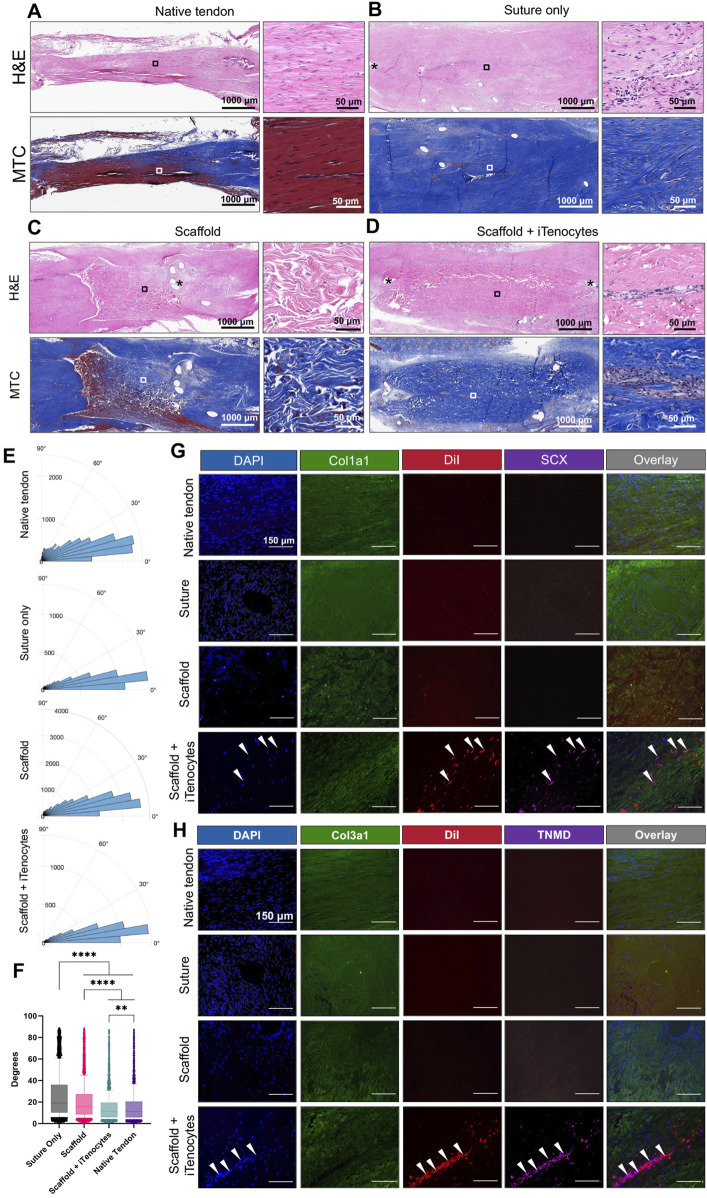
Histology and immunofluorescence of Achilles tendon defects *in vivo*. Representative H&E and MTC-stained sections of native tendon tissue **(A)**, tendon defects treated with suture only **(B)**, scaffolds only **(C)**, or iTenocyte-seeded scaffolds **(D)**. Rose plots of the nuclei orientation **(E)**. Statistical analysis of the variability of the nuclei orientations **(F)** Representative images of native tissue as well as tendon defects treated with suture only scaffold, or iTenocyte-seeded scaffold. DAPI (blue) served as a nuclear staining and Cy2-conjugated antibodies against collagen 1 and 3 was used to visualize structural collagen within native tissue, and suture-treated animals as well as implanted scaffolds (green). DiI labeled cells (red) as well as Cy5-conjugated tenocyte markers SCX and TNMD (pink) were only observed in tendon defects treated with iTenocyte-seeded scaffold **(G, H)**. Scale bars = 150 µm **p < 0.01, ****p < 0.0001.

Representative histological Hematoxylin Eosin (H&E) and Masson’s trichrome (MTC) stains showed a well-organized structure of the native tendon tissue (Figure 8A), whereas tendon defects treated with suture only display a highly disorganized connective tissue (Figure 8B). Interestingly, the tendon defects repaired with unseeded scaffolds also revealed a very disrupted structure with no detection of cellular penetration (Figure 8C). On the other hand, iTenocyte-seeded scaffold-repaired defects showed a more compact structure with a clear lining of initially implanted cells. A cellular alignment appears in the center of the scaffold, even 9 weeks after implantation, probably caused by the implantation technique in which the scaffold wrapped around the defect when the cell-seeded mostly surface is on the inside of the scaffold, forming a tube-like structure (Figure 8D).

The nuclei orientation was measured to approximate the linearity of the collagen fibers in the tendon tissue. We analyzed the H&E-stained sections and collected angle measurements of the nuclei for each of the four treatment groups (Figure 8E). The variability in angle measurements of the nuclei were compared across the four groups using the Kolmogorov-Smirnov Test. Statistically significant findings (*p*-value < 0.01 or *p*-value < 0.0001) (Figure 8F) were obtained when comparing the variability of one group to each of the others indicating a difference in the linearity of the collagen fibers across the different treatments. The nuclei variability from least to most variable corresponded to Native tendon, Scaffold + iTenocytes, Scaffold, and Suture only.

Immunofluorescent staining further demonstrated a more regular tissue formation in Achilles tendon defects treated with iTenocyte-seeded scaffold when compared to such defects treated with suture or scaffold only (Figure 8). Although antibodies against human tendon markers were used, some cross reactivity with rat proteins was expected.

Antibodies against Col1a1 and Col3a1 were used to visualize these matrix proteins in native tendon tissue and suture only-treated animals as well as with the scaffolds with or without cells. Of interest, cells positive for SCX and TNMD, which are proteins crucially involved in tenocyte proliferation, were only detected within iTenocyte-seeded collagen scaffolds.

## 4 Discussion

The present study explored the potential of iTenocyte-seeded collagen scaffolds to enhance the tissue regeneration and biomechanical functional recovery of tendon issue in rats after a critical size defect in the Achilles tendon.

Initial assessment of biomechanical parameters of wet DuRepair™ collagen scaffolds revealed a comparable young’s modulus, maximum force, maximum displacement, and stiffness compared to native rat tendon tissue. For a biomaterial to be utilized for tendon defects, it is of crucial importance that its biomechanical properties, with or without cells, match the properties of the target tissue as much as possible (Green and Elisseeff, 2016; Miramini et al., 2020; Hollister et al., 2002). Since healthy tendon tissue needs to transmit forces between muscle and bone with minimal deformation or loss of energy, other research groups have stressed the importance of matching properties between implantable biomaterials and native tendon tissue (No et al., 2020; Yang et al., 2013). Due to the overall importance of the material’s surface structure, we investigated the topography of DuRepair™ collagen scaffolds by means of SEM. Our findings show a relatively rough and porous structure, both on the surface and center zones of the biomaterials. Not only do pores play a crucial role in providing cellular attachment opportunities, but the surrounding interconnecting structure further allows cells to migrate and attach onto the scaffold. Based on these findings, the DuRepair™ collagen scaffold was considered a promising biomaterial for the intended regeneration of tendon defects.

In a second set of experiments, we determined the optimal seeding density and culture duration for collagen scaffold seeded with iTenocytes. We could show that there was no benefit of seeding biomaterials with more than 500,000 iTenocytes per the intended for implantation scaffold size. The resulting plateau of cellular retention between 500,000 and 1,000,000 cells may have been a result of overseeding or overcrowding. Other research groups have seen identical effects of cell loss secondary to crowding, which resulted from negative effects on nutrient delivery and, consequently, reduced metabolic activity, leading to reduced cellular retention (Dudas et al., 2008; Wang T. et al., 2013). Hence, we seeded collagen scaffolds with 500,000 iTenocytes for a total of 3 days *in vitro* prior to implantation into rat Achilles tendon defects.

Following cellular seeding of collagen scaffolds, we analyzed the seeding depth by means of histological H&E-staining and surface topography via SEM which revealed attachment of the cells on the biomaterials’ surface. The scaffolds that were seeded after 4 h had been freshly seeded viable cells, making the top layer to be purple due to the cell nuclei (Figures 5A, B). On the other hand, the scaffolds that were seeded for 3 days had cell death due to lack of cell adhesion or penetration onto the scaffold. Since the scaffold was also in culture longer, the scaffold had more cellular and media interaction, causing it to appear pinker in the histology images (Figures 5C, D). Since cells are thought to be located within the center of a tube-like structure following defect repair by wrapping the biomaterial around the tendon ends, the seeding depth into the material itself only represented a secondary parameter.

For this study, gait analysis was used to assess the degree of functional recovery overtime of Achilles tendon defects repaired with suture only, scaffold only, or scaffolds seeded with 500,000 iTenocytes over time. Our data shows between the three treatment groups, rats treated with iTenocyte-seeded collagen scaffolds were the only ones with a right paw width comparable to baseline as early as week 6. At 9 weeks post injury, all treatments groups showed an improved paw width. The iTenocyte-seeded scaffold and suture only treatment groups had improved right heel length and right foot length at 6 weeks post injury. Furthermore, both right foot length and heel length of animals treated with iTenocyte-seeded collagen scaffolds showed an accelerated recovery when compared to those animals treated with sutures or scaffolds only.

When the triceps surae muscles exert their forces throughout the Achilles tendon into calcaneus, it provides leverage and causes plantarflexion to change from a lift off and lowering position into the flat position. Rat tendon injury experimental models have shown the effectiveness of using the footprint test because it demonstrates configuration during the supination of the foot in the closed chain mode. The tendon injury leads to a heel drop and dorsiflexion of a plantarflexed foot, which causes a morphologically longer and thinner foot in a rat model (Murrell et al., 1993). Also, deformity of foot and heel length is based on the involvement of dorsal intrinsic muscles, which abduct and extend the toes in both rodents and humans. The triceps surae also form corporations with planar fascia through proximal attachment on the calcaneus, which causes the ankle joint to force the metatarsophalangeal joints into a plantar flexion position similar to the position observed in humans (Rodgers, 1995; Fraser et al., 2016). Simultaneously, plantar intrinsic abductor muscles are inserted into the calcaneus, abducting the hallux and flexing toes. This, in turn, contributes to foot stabilization and eccentric control of the planar descent during loading (Fraser et al., 2016). Both the calcaneus drop and intrinsic muscle function improvement lead to a positive dynamic of foot length measurement, which can be seen in all groups.

Of interest, the overall regenerative progression of this model followed the same healing patterns as previously observed by our research group, indicating a reliable standardization of the animal model used (Kaneda et al., 2023). We can conclude a positive trend in the functional improvement of muscles biomechanics during the recovery process. As the muscles wrap around into the foot at different points and work against one another, we report a synergistic functional effect on the positions of the joints and ligaments in both hind limbs, plantar and toe splay control, metatarsophalangeal extension, forefoot stabilization to gain back the symmetry of quadrupedal trot.

Although the injury was on the right side, most of the paw angle change was seen on the left side. We can speculate that the right paw was injured and treated via suture, scaffold or iTenocyte seeded scaffold, the paw remained at one certain position. This change in foot structure caused the uninjured (left) paw to obtain more body weight to be placed onto the paw and change its locomotion, causing the variability.

The positive changes in the right paw configuration of the iTenocyte-seeded scaffold at week 6 (which reflects in the right paw width graph) resulted in an increased angle in the left paw at week 6 (which reflects in left paw angle graph). There is a correlation between paw width configuration and paw angle in both feet. The scaffold only had the effect of stabilizing the ankle-joint complex.

Biomechanical testing of excised tissues at the end of the 9-week experiment revealed that both iTenocyte-seeded scaffold and scaffold only-treated rats demonstrated improved biomechanical properties compared to rats treated with suture only. Achilles tendon defects treated with suture only also exhibited the lowest recovery potential. The iTenocyte-seeded scaffold and scaffold only groups had no significant differences in biomechanical properties. This may be explained by insufficient cell adhesion onto the scaffold. This demonstrates that the scaffold itself can provide the biomechanical support, however the cells are needed for functional repair. The beneficial effect of iTenocyte-seeded scaffolds may arise from the alignment of the seeded cells within the center of the tube-like scaffold following implantation, leading to a guided alignment of SCX-overexpressing cells alongside the central part of the scaffold.

To gain further insight in the cellular behavior after biomaterial transplantation, we analyzed histological and immunohistochemical sections. The native tendon tissue has an aligned collagen fibers with thin cell nuclei, while the injured tendon tissues, especially the suture only treatment group, has areas of cellular and collagen disarray similar to the study done by Scott et al. (2011). iTenocyte-seeded scaffold demonstrated the most organized tendon tissue when compared to suture only and scaffold only groups. The iTenocyte-seeded scaffold-treated tendons’ histology showed a clear line of cells within the center of the scaffold, indicating the long-term survival and the contribution of the iTenocytes to the tissue repair. This alignment may be the result of cells being trapped within the inside the scaffold following implantation, ultimately leading to a guided growth alongside the scaffold center. The general tissue orientation analysis also showed advantage of the cell-seeded scaffold to other groups (Figures 8E, F). Immunohistochemical staining provided visual confirmation backing up our initial interpretation based on the assessment of H&E-stained and MTC stained sections. Indeed, only animals treated with iTenocyte-seeded scaffolds exhibited cells labeled with DiI, which can therefore be traced back to the implantation origin. Both Collagen1 and Collagen 3 are two matrix genes for tenogenic differentiation that play a strong role in development and healing of tendons, we expect to see Collagen 1 and 3 in the iTenocyte-seeded scaffold since they were the only group with human samples, but this was not seen in our immunohistochemistry. All of our samples had Collagen 1 and 3, which could have been due to antibodies reacting to the Nude rat and collagen scaffold. For the scaffold and iTenocyte seeded scaffold, the areas with no collagen matrix proteins are the scaffold’s pores. Moreover, the cells seemed to be predominantly located in the center of scaffolds that were wrapped around the two remaining ends of tendon tissue following the creation of a 3 mm defect. However, further penetration into the scaffolds did not occur after the implantation of the biomaterial, restricting the seeded cells to only be able to proliferate and differentiate on the scaffold surface.

This study is not without limitations. The Nude rats used within this experimental setup were primarily chosen to avoid immuno-rejection of the human cells. However, they lack the immune system interaction with the implanted cells and the lack of natural immune system may affect the healing process. There is some evidence in our previous study that iPSC-derived cells will avoid immune response also and rejection in immunocompetent animals (Später et al., 2023). However, this should be further tested in a tendon context, which is less immunoprivileged site than a joint or intervertebral disc. Moreover, the anatomical dimensions are significantly different to that expected in humans. This discrepancy hinders a standardized evaluation of a potential clinical success (Bottagisio and Lovati, 2017; Hast et al., 2014; Thomopoulos et al., 2015). Hence, following this proof-of-concept study, larger animal models that mimic the human anatomical structure more closely, may be utilized in future studies.

Secondly, the population of the scaffold by the cells was not ideal. We were able to show an optimal seeding density and incubation time for iTenocytes on DuRepair™ scaffolds. Although cells illustrated an adequate survival and cellular attachment, cells were not able to penetrate the scaffold due to its pore sizes. After wrapping the scaffold around tendon tissue surrounding the 3 mm defect, cells were trapped on the inside of the resulting tube-like structure. Even though cells successfully aligned within the center of the scaffolds, it may be beneficial to find another way to properly seed the biomaterial or to find another collagen biomaterial that will allow for efficient cell attachment to further enhance the healing capacity and recovery speed of treated defects.

## 5 Conclusion

Overall, DuRepair™ collagen scaffolds alone or seeded with iTenocytes has significantly enhanced the biomechanical properties of ruptured Achilles tendon tissue and directionality of the tendon fibers in rats. Seeding the scaffold with iTenocytes improved the gait function of the repaired tendons. The findings from this study represent a steppingstone on the way to future preclinical studies and clinical treatments of patients.

## Data Availability

The raw data supporting the conclusions of this article will be made available by the authors, without undue reservation.
