# Supplementary material for: Collagen scaffold-seeded iTenocytes accelerate the healing and functional recovery of Achilles tendon defects in a rat model
Source: Front Bioeng Biotechnol. 2024 Dec 6;12:1407729. doi: 10.3389/fbioe.2024.1407729 (PMC11658981; doi:10.3389/fbioe.2024.1407729)
Supplement: Supplementary file 1 [file DataSheet1.docx]

Supplementary Material

# Supplementary Figures and Tables

##
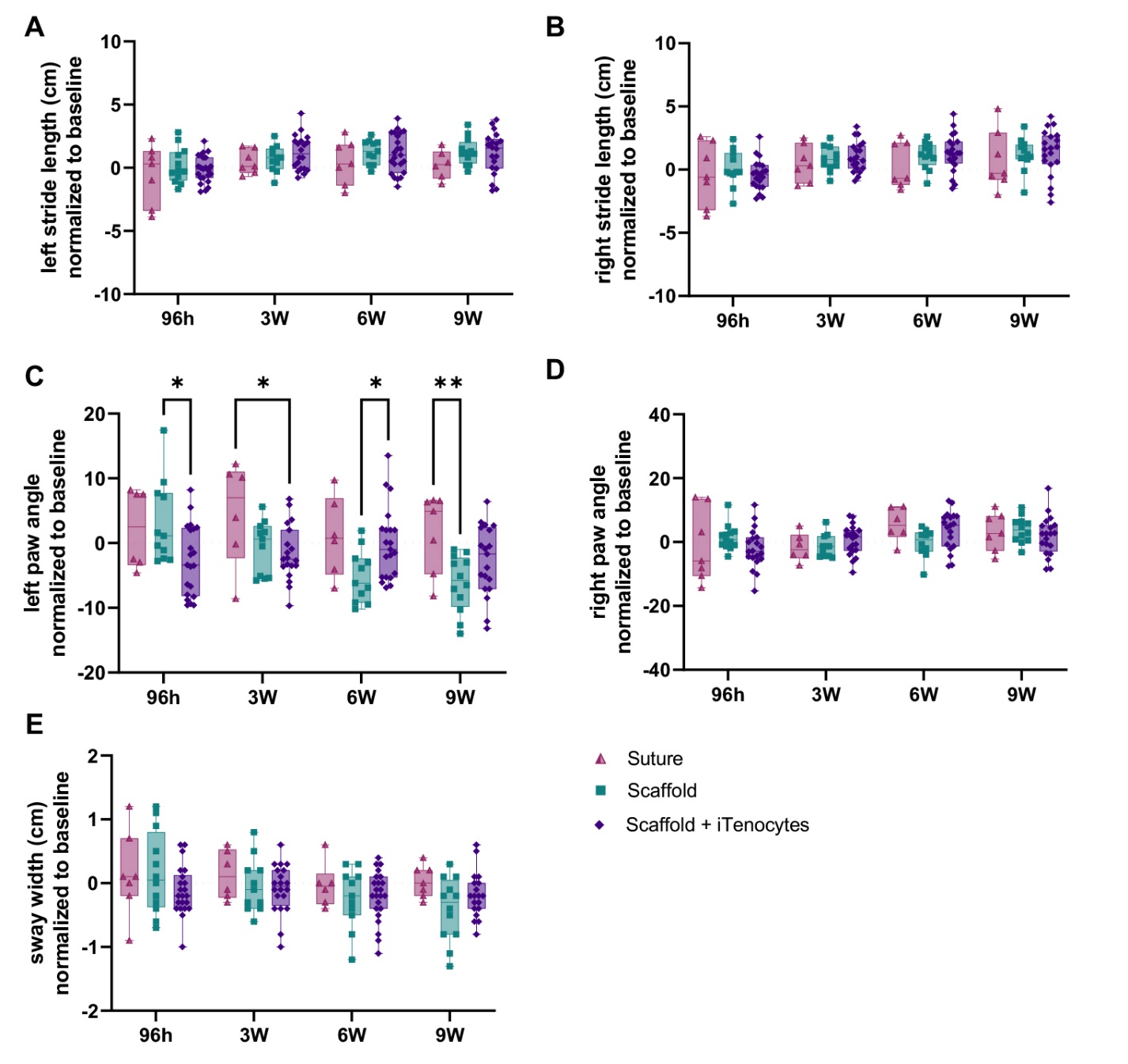
Supplementary Figures

**Supplementary Figure 1.** Gait functionality of Achilles tendon defects treated with iTenocyte-seeded collagen scaffolds *in vivo*. Stride lengths of left (A) and right (B) paws, angles of left (C) and right (D) paws and sway width (F) on 96 hours, week 3, week 6 and week 9 after injury and treatment. ^*^p < 0.05, ^**^p < 0.01. Suture only n=8; scaffold only n=12, scaffold with iTenocytes n=12.

## Supplementary Tables

| **Type** | **Antigen** | **Conjugation** | **Host** | **Target** | **Manufacturer** | **Catalog #** | **Dilution** |
| --- | --- | --- | --- | --- | --- | --- | --- |
| 1° | SCX | - | Rabbit | Human | Invitrogen | PA5-115874 | 1:100 |
| 1° | Col3A1 | - | Goat | Human | US Biological | C7510-44K | 1:100 |
| 1° | Col1A1 | - | Goat | Human | Biorad | 131001 | 1:100 |
| 1° | Tenomodulin (TNMD) | - | Rabbit | Human | Sigma Aldrich | HPA055634 | 1:100 |
| 2° | IgG (H+L) | Cy2 | Donkey | Goat | Jackson ImmunoResearch | 705-225-147 | 1:200 |
| 2° | IgG (H+L) | Cy5 | Donkey | Rabbit | Jackson ImmunoResearch | 711-175-152 | 1:200 |

**Supplemental Table 1**: List of antigens, host, targets, conjugations, and dilution used for the immunohistochemistry.

| **Gene** | **Abbreviation** | **Species** | **PubMed RefSeq** | **Catalog Number** |
| --- | --- | --- | --- | --- |
| 18S | - | Human | - | HS03003631_g1 |
| Scleraxis | SCX | Human | NM_001080514.2 | Hs03054634_g1 |
| Collagen I | Col1 | Human | NM_000088.3 | HS00164004_m1 |
| Tenascin | TNC | Human | NM_002160.3 | HS01115665_m1 |
| Mohawk Homeobox | MKX | Human | NM_001242702.1 | HS00543190_m1 |
| Decorin | DCN | Human | NM_001920.4 | HS00370384_m1 |
| Tenomodulin | TNMD | Human | NM_022144.2 | Hs00223332_m1 |
| Thrombospondin 4 | THBS4 | Human | NM_001306212.1 | Hs00170261_m1 |
| Collagen 3 | Col3 | Human | NM_000090.3 | HS00943809_m1 |
| Transforming Growth Factor, β1 | TGFβ1 | Rat | NM_021578.2 | RN00572010_m1 |
| Collagen 10 A1 | ColXa1 | Rat | XM_001053056.6 | Rn01408030_m1 |

**Supplemental Table 2**: List of primers used for gene expression analysis
